# Being More Educated and Earning More Increases Romantic Interest: Data from 1.8 M Online Daters from 24 Nations

**DOI:** 10.1007/s12110-022-09422-2

**Published:** 2022-04-05

**Authors:** Peter K. Jonason, Andrew G. Thomas

**Affiliations:** 1 Department of General Psychology, University of Padua, Via Venezia, 12, 35131 Padua, Italy; 2 University of Cardinal Stefan Wyszyński, Warszawa, Poland; 3 Swansea University, Swansea, United Kingdom

**Keywords:** Mate choice, Sex differences, Education, Income, Cross-cultural analysis, Online dating

## Abstract

How humans choose their mates is a central feature of adult life and an area of considerable disagreement among relationship researchers. However, few studies have examined mate choice (instead of mate preferences) around the world, and fewer still have considered data from online dating services. Using data from more than 1.8 million online daters from 24 countries, we examined the role of sex and resource-acquisition ability (as indicated by level of education and income) in mate choice using multilevel modeling. We then attempted to understand country-level variance by examining factors such as gender equality and the operational sex ratio. In every nation, a person’s resource-acquisition ability was positively associated with the amount of attention they received from other site members. There was a marked sex difference in this effect; resource-acquisition ability improved the attention received by men almost 2.5 times that of women. This sex difference was in every country, admittedly with some variance between nations. Several country-level traits moderated the effects of resource-acquisition ability, and in the case of unemployment this moderating role differed by sex. Overall, country-level effects were more consistent with evolutionary explanations than sociocultural ones. The results suggest a robust effect of resource-acquisition ability on real-life mate choice that transcends international boundaries and is reliably stronger for men than women. Cross-cultural variance in the role of resource-acquisition ability appears sensitive to local competition and gender equality at the country level.

Although romantic relationships may seem trivial compared with other matters in the modern world, such as pandemics, global climate change, and food insecurity, we would not be here to ponder these modern-day existential threats if our ancestors had made deleterious mate choices. Mate choice, in both our ancestral and modern past, has a real impact on individual well-being. Mating mistakes have both reproductive and psychosocial outcomes, including depression and suicide after a breakup and anxiety in response to romantic failures (Hewitt et al., 2012; Kõlves et al., 2011; Rhoades et al., 2011). Given the importance of finding a romantic partner and the benefits provided (e.g., psychological health, finances, and reproduction), entire industries have tried to capitalize on people’s desires to form fruitful and healthy relationships. These have evolved from singles ads in newspapers 100 years ago to online dating over the past 20 years.

Online dating websites and applications are quickly becoming the primary method of meeting potential partners in the Western world (Rosenfeld et al., 2019), and thus greatly influence how we identify and choose potential suitors. For example, they present users with an almost inexhaustible number of potential partners that can create choice-overload (D’Angelo & Toma, 2017). One method of coping with so many choices is to filter them by one’s necessities (Li et al., 2002, 2013; Thomas et al., 2020).

Beyond physical attractiveness—an initial and well-studied filter (Egebark et al., 2021; Kenrick et al., 1993), another fundamental and cross-culturally important feature that people use to identify suitors is the ability to acquire resources—what might be called “competence” (Jonason & March, 2021)—which will, in part, be indicated by individual differences in a person’s ability to acquire resources for themselves, their offspring, and their partner and family. According to UNICEF, raising a child from birth to 17 years of age costs between US$900/year in developing countries and US$16,200/year in developed countries.^1^ In 1800, around 50% of children in the United States died before the age of five.^2^ Gaining sufficient resources to create and rear offspring is a universal problem that exists for most mammals, humans included, and finding a partner who can help may be an essential part of mate selection. People, today or yesterday, rarely know for sure whether a potential suitor has this ability so they may seek cues that act as proxies, such as level of education and income (Egebark et al., 2021; Hopcroft, 2021; Jonason & Antoon, 2019; Jonason et al., 2012). Higher IQ or education enhances desirability in self-report (Prokosch et al., 2009), speed dating (Kurzban & Weeden, 2005), census data (Hopcroft, 2021), and personal ads (Pawlowski & Koziel, 2002) along with experimental (Buunk et al., 2002; Egebark et al., 2021) and online dating (Egebark et al., 2021; Lin & Lundquist, 2013) studies. And, importantly, more education and income at the individual and country levels lead to less childhood mortality, especially when the increase occurs in women (Collison et al., 2007; Gakidou et al., 2010; Hobcraft et al., 1984; Strulik, 2004). Increased education and income may lead to lower childhood mortality because children are not engaged in labor, parents experience fewer existential threats, better healthcare is provided, and parents are freer to invest more time in care. In ancestral environments, individuals were likely to have lived in a constant state of threat from predators and uncertainty regarding where the next meal might come from. Those individuals who were better able to solve these challenges through their intelligence and ability to acquire resources would have had greater reproductive success than their peers. Those who had preferences for partners with such qualities would have similarly had more reproductive success and, insomuch as these abilities and preferences are heritable, any offspring would also have fared better than those born to parents indifferent to the resource-acquiring abilities of their partners. Over generations, this process of selection might be (in part) responsible for the value placed on resource-acquisition ability in the modern mating market.

Despite the substantial body of research on mate preferences and choice, some persistent problems with this research warrant addressing. Researchers often rely on “cold” judgments wherein participants report their hypothetical interest in romantic partners (DiPrete & Buchmann, 2006; Gignac et al., 2018). This reveals what people *think* they want, but not necessarily who they *choose*, so-called hot judgments. Studies relying on hot judgments—such as those relying on speed dating—may suffer from issues of statistical power, cultural specificity, and sampling bias (Egebark et al., 2021; Kurzban & Weeden, 2005; Li et al., 2013). Ecological validity aside, a common issue for research on mate choice is that it is almost exclusively relies on monocultural samples, raising questions as to the generalizability of their findings. When researchers do attempt to sample cross-cultural trends, the researchers appear motivated to simply create a list of what people desire rather than to understand specific aspects of mate choice (Buss, 1989), or they are pragmatically forced (e.g., by sample size issues) to reduce cross-cultural comparisons to East-West differences (Thomas et al., 2020). Despite the contention of sociocultural psychologists that culture plays a major role in determining mate choice (Eagly, 1987; Eagly & Wood, 1999), little research has examined variance from country to country. To address these matters and better understand the role of resource-acquisition ability in mate choice, we leverage behavioral data from an online dating service that operates in two dozen countries.

The idea that higher resource-acquisition ability may increase someone’s desirability as a mate is not a controversial one. Although the desirability of more-educated and intelligent people may not be strictly linear (Jonason & Antoon, 2019; Jonason et al., 2019), having more of a desirable trait (regardless of the relative position of the rater) should lead to more desirability given basic microeconomics assumptions (Egebark et al., 2021; Hopcroft, 2021). However, little research has quantified the magnitude of this relationship around the world. Given the role of resource-acquisition ability in mate choice and childhood mortality, understanding the role of these factors in mate choice seems important. It may better inform campaigns to reduce childhood mortality and also improve people’s efforts to find satisfactory or satisficing romantic partners. Therefore, our first prediction is that increased resource-acquisition ability should enhance the attention people receive in response to their online dating profile.

While most agree that more resource-acquisition ability is valuable, considerable debate exists around the importance men and women might assign to this feature (Egebark et al., 2021; Hopcroft, 2021). Simple economic models (e.g., loss aversion) of mate choice fail to differentiate between the sexes because they assume a monomorphic sexual psychology (Jonason et al., 2020). In the psychology of mate choice, two primary epistemologies make widely divergent predictions about the role of resource-acquisition ability in mate choice. Sociocultural researchers claim that intelligent women face a backlash (Eckes, 2002; Egebark et al., 2021) because they have a sex-role-violating trait, which leads to less likability (Szymanowicz & Furnham, 2011); in contrast, men who have more resource-acquisition ability are rated more favorably (Egebark et al., 2021; Fiske et al., 2002; Hopcroft, 2021), suggesting that it leads to less desirability as romantic partners for women and more desirability for men. If this framework is correct, we should find a crossover interaction between resource-acquisition ability and participant’s sex in the amount of interest received in online dating—increased resource-acquisition ability should enhance the interest received by men and diminish that of women.

In the second framework, instead of looking to culture for explanations of sex differences, evolutionary scholars look to biology, drawing on parental investment theory (Trivers, 1972). Although in some modern contexts there have been attempts to equalize investment in offspring, women continue to invest more in their offspring than men do (Maume, 2008), much of which is a function of asymmetries in minimum obligatory investment generated by conception and gestation costs. Because female mammals must face the costs of internal gestation, and many, including humans, raise children alone and have done so over evolutionary time, there may be sex differences in mate choice (Buss & Schmitt, 1993; Thomas et al., 2020). In the case of resource-acquisition ability, women may seek this quality in their male partners to help support their gestation and rearing costs (Jonason et al., 2012). That is, ancestral women who emphasized resource-acquisition ability in their partners will have had an easier time reproducing because their partners (1) could provide them with food when their gathering abilities decrease thanks to pregnancy and rearing and (2) could provide food directly to their offspring. Today this translates into several effects. For instance, women ascribe more importance to a mate’s intelligence and income than men do (Buss et al., 2001; Hopcroft, 2021; Souza et al., 2016), and their interest in this feature holds across relationship durations (Jonason & Antoon, 2019; Jonason et al., 2019). From this perspective, both sexes would have benefited from choosing a partner who could acquire resources because it benefits their mutual offspring, but it is women who will benefit from this more than men (Hopcroft, 2021; Prokosch et al., 2009). Taken together, if this approach is correct, both sexes should receive greater attention on online dating platforms if their resource-acquisition ability is high, but this effect should be larger for men than for women.

Although considerable research on mate choice has examined sex differences (Buss & Schmitt, 1993; Li & Metzler, 2015), less has examined cross-national variability (Gangestad et al., 2006; Kasser & Sharma, 1999). This may be for several reasons. First, the availability of data on mate choice (as opposed to mate preferences) around the world is limited and rarely standardized from nation to nation. Second, most studies on mate choice rely on “small” data with experimental methods. Third, researchers may not have developed sufficient theoretical cause to invest in the collection of cross-national data, which until recently was exceedingly difficult to undertake. Nevertheless, there is reason to believe that understanding the role of “culture” in mate choice may be warranted. For instance, gender equality has been suggested as an important feature of sex differences in mate preferences (Eagly & Wood, 1999), and traits such as chastity are important only in some countries (Buss, 1989; Thomas et al., 2020).

To better understand nation-level differences in the impact of resource-acquisition ability on one’s desirability as a romantic partner, we considered four variables: national wealth, gender equity, the operational sex ratio, and unemployment.^3^ One of the main features of Westernization/modernization is the increased importance of education and income in people’s lives and careers. We would therefore expect more-developed nations (e.g., less unemployment, more gender equality, and greater income) to have people who more highly value resource-acquisition ability in their romantic partners. That is, as cultures place a greater emphasis on education and income (as opposed to other sources of status and resources), mate choice mechanisms in people should be sensitive to the change. This is especially true if people calibrate their mate preferences to modern contexts, as suggested by sociocultural psychologists (Eagly, 1987). However, the value placed on resource-acquisition ability in mate choice might be moderated by the sex of the person.

Evolutionary and sociocultural researchers agree that variation in local contextual factors can affect the magnitude and direction of sex differences in mating psychology (Eagly & Wood, 1999; Schmitt et al., 2017). We explore the role of several nation-level variables to account for cross-national differences in sex differences in the power of resource-acquisition ability to lead to greater interest in the online dating context. For instance, traditional sex roles, and their related mate preferences, may amplify sex differences by reinforcing gender stereotypes. If so, we would expect societies marked by greater gender equality to have smaller sex differences in the benefits accrued by being seen as more competent. Alternatively, it might be that more modern countries have less competition for resources and thus may set up a context where the sexes can more freely express their dispositional tendencies in safety (Pollet & Nettle, 2008; Watkins et al., 2012).

In this article, we present the first study on actual mate choice to use data from nearly 1.8 million users of an international, online dating company operating in 24 countries. We focus on sex differences in the amount of romantic interest received from online daters and the role of combined education and income (i.e., resource-acquisition ability) in accounting for individual differences in interest received. We then examine country-level variance in interest received by men and women of differing levels of resource-acquisition ability with data on the gross national income per capita, gender development (GDI, discussed below), the operational sex ratio, and level of unemployment. This undertaking constitutes one of the most wide-scale tests of the predictions from sociocultural and evolutionary models of mate choice and sex differences to date on the matter of resource-acquisition ability.

## Methods

### Participants

Data for this project were provided by the Spark Networks Services GmbH (formerly Affinitas), which operates in more than 20 countries under different names (e.g., EliteSingles, eDarling). Members of the sites are single adults looking for a long-term, committed relationship. They are predominantly heterosexual (96%). The company provided as much data for each country as possible through Excel files, with the largest samples (USA, Germany, France) containing membership records for more than 1 million people. In total, the sample exceeded 9.5 M. The transferred data lacked personal details (e.g., name, email) or specific job labels (asked in free text) that could be used to identify members.

The data were thoroughly cleaned before analyses to remove potentially bogus and inactive membership records. Specifically, we excluded the records of members who had missing data for the key variables of interest, were over the age of 80, had not accessed their account within the past three years, and were outliers for number of logins and self-reported height. Lastly, we excluded any members who did not visit anyone else’s profile and/or had no visitors of their own. The final dataset consisted of just over 1.8 M records. Our data screening process was conservative, but we felt that this was justified (1) to give confidence that our dataset contained active, genuine members of the dating websites and (2) given the substantial number of people in each country. Approval for the secondary data analysis was received by the first author from the ethics board at Western Sydney University. Data for this project are the property of the Sparks Network; however, shared summary and country level data are available via OSF.^4^.

### Measures and Statistical Analysis

Each member’s record contained dozens of variables, ranging from height to personality to religion. For brevity, we mention here only those that we included in our analyses. To capture how much attention each profile received, we created a composite variable called IOI (Indicators of Interest). This variable was formed by combining the number of messages, “likes,” and “winks” a member had received from others (Cronbach’s α = 0.72). To predict IOI, we used the member’s country of residence, sex, and their resource-acquisition ability. Resource-acquisition ability was measured by combining (*r* = .32; *M* = 7.75, *SD* = 2.48) the member’s income (1 = *Very low*; 7 = *Very high* [based on local currency]) with their level of education (1 = *No High School Degree*; 7 = *Doctorate* [e.g., MD, PhD, JD]) consistent with work suggesting education and intelligence may operate as resource-acquisition ability markers (Fletcher et al., 1999; Jonason & March, 2021).^5^

We used a multilevel negative binomial model for the analysis to account for the fact that IOI followed a count distribution and to allow our intercept and slopes to vary across countries. In the null model, we included only random intercepts for country and control variables. Because older and more active accounts might receive more attention than new and inactive accounts, we included account age and length of time since last log-in as control variables in the analysis. Some of the websites required members to upgrade to a “premium” account to use certain functions (e.g., to send and receive messages rather than just “like” and “wink”). Although the number of premium account holders was small (2.8%), we decided to include account status as a control variable because account status could feasibly influence the number of IOI received. It could, for example, give the impression that a member is “serious” about online dating because they are willing to invest money.

Our second model added sex, resource-acquisition ability, and their interaction. To avoid producing an overly complex model, we used 10% of the sample to test whether allowing the slopes of sex and resource-acquisition ability to vary revealed variation across countries. This preliminary analysis revealed a good amount of variation for sex (Var.Comp = 0.16, *SD* = 0.40) but not resource-acquisition ability (Var.Comp < 0.01, *SD* = 0.03). Thus, in our main analysis we opted to allow random slopes for sex only.

Lastly, to examine possible determinants of country-level variance in the influence of resource-acquisition ability on dating profile interest, we added level 2 (country-level) measures to the second model and allowed them to interact with our level 1 variables of sex and resource-acquisition ability. We produced a separate model for each country-level variable because of the small number of countries included in it. The four country-level variables were the 5-year average of its operational sex ratio (ages 15–65; OSR); Gross National Income (GNI); percent of the population not in education, employment, or training (NEET) from the World Development Indicators;^6^ and its 2018 Gender Development Index (GDI) value from the Human Development Report.^7^

## Results

**Fig. 1:**
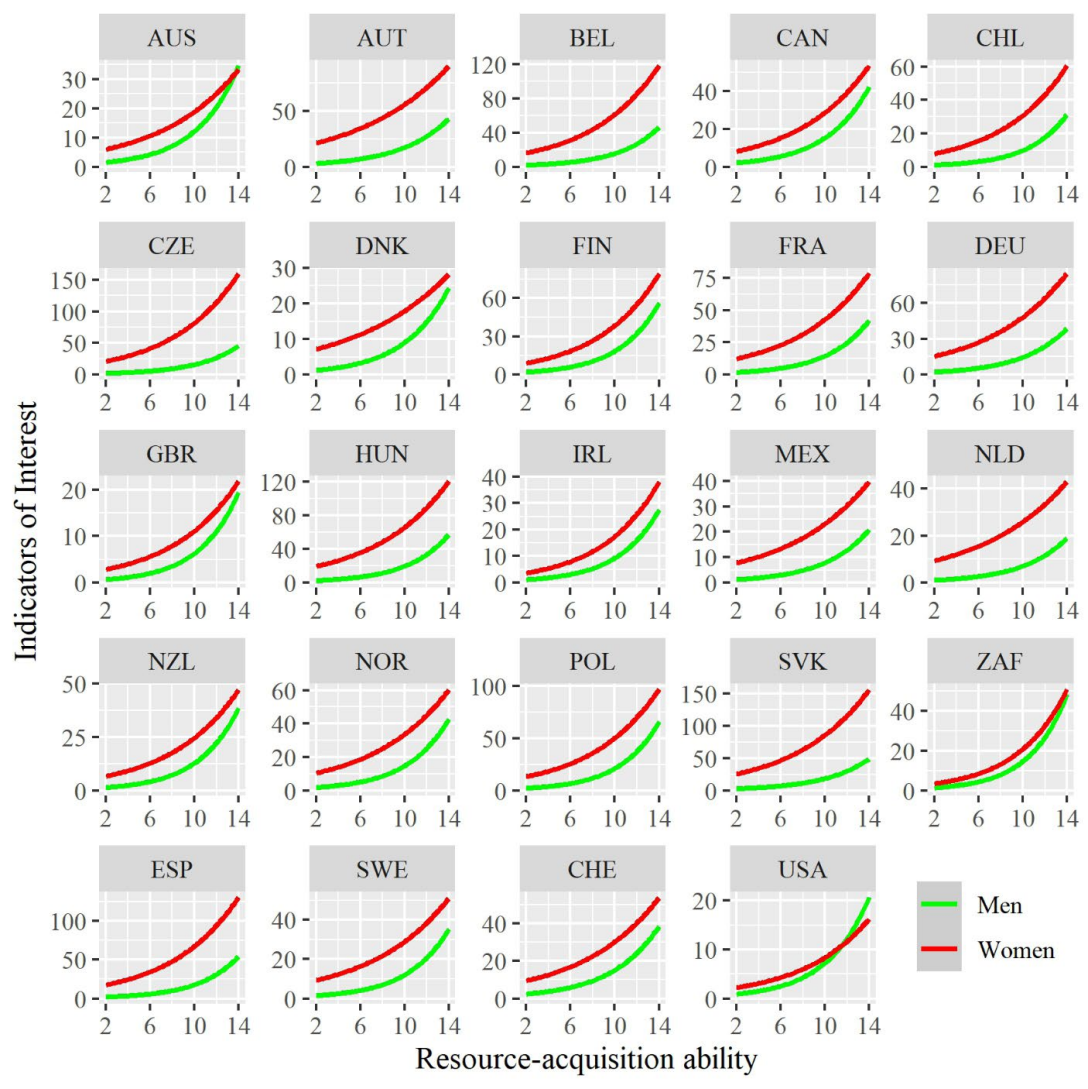
The predicted impact of resource-acquisition ability on the number of indicators of interest (IOI) a dating profile received. Predictions are separated by country and by sex. Ribbons showing 95% confidence intervals are present but imperceivable

As expected, given the sample size, all predictors and interactions for the null model and the model containing just sex and resource-acquisition ability were significant (*p* < .001). Thus, in Table 1 we rely on confidence intervals to evaluate the impact of each predictor. The analysis revealed that premium account status, recent account activity, and having an older account were all positive predictors of IOI. In terms of our key predictors of interest, resource-acquisition ability showed a positive relationship with profile interest, a pattern that was consistent across all countries (Fig. 1). There was a substantial sex difference in attention; woman received between 540% and 780% more IOI than men. Importantly, there was an interaction between resource-acquisition ability and sex. This relationship was negative, suggesting that the attention-enhancing effect of resource-acquisition ability was smaller for women than for men.

**Table 1:** Results of a negative binomial mixed effects model predicting indicators of interest using account status/activity, sex, and resource-acquisition ability. The intercept and slope for sex was allowed to vary by country

*Fixed effects*	*B*	*SE*	exp(*B*)	95% CI	
Intercept	−0.37	0.05	0.69	0.62	0.77
Premium account status†	1.01	0.01	2.73	2.70	2.77
Time since last login†	−0.26	<0.01	0.77	0.77	0.78
Account age	0.66	<0.01	1.93	1.92	1.93
Sex [Men = 0; Women = 1]	2.02	0.08	7.52	6.41	8.81
Resource-acquisition ability	0.25	<0.01	1.29	1.29	1.29
Sex × Resource-acquisition ability	−0.11	<0.01	0.89	0.89	0.90
*Random effects*	Var.comp	*SD*			
Intercept	0.07	0.27			
Sex	0.16	0.40			
*Model fit*	AIC	BIC			
	11,371,820	11,371,956			

† = standardized

To better understand this interaction, we used the model to generate estimated marginal means at varying levels of resource-acquisition ability (which controlled for premium account status). Men with a resource-acquisition ability 1 *SD* greater than the mean received 255% more IOI (*M* = 15.48, *SE* = 0.84) than those 1 *SD* less than the mean (*M* = 4.36, *SE* = 0.24; *p* < .001). In contrast, women with a resource-acquisition ability 1 *SD* greater than the mean received 103% more IOI (*M* = 36.79, *SE* = 4.06) than those 1 *SD* less than the mean (*M* = 18.11, *SE* = 2.00; *p* < .001).

**Fig. 2:**
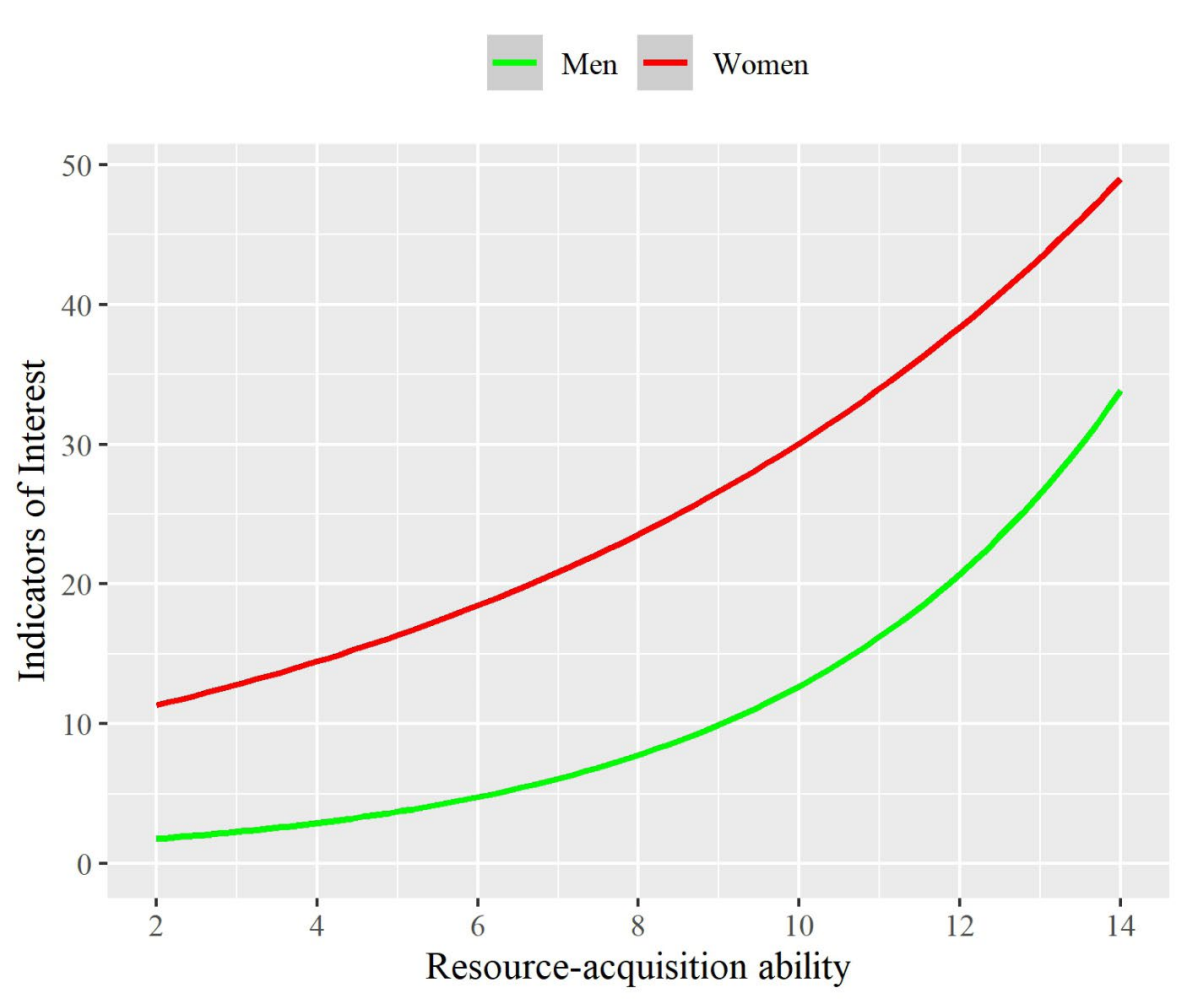
The predicted impact of resource-acquisition ability on the number of indicators of interest (IOI) a dating profile received, separated by sex. Ribbons showing 95% confidence intervals are present but imperceivable

Interestingly, the large sex difference in IOI received regardless of resource-acquisition ability (*M*_Women_ = 25.81, *SE* = 2.85 vs. *M*_Men_ = 8.21, *SE* = 0.45; *p* < .001) causes a visual illusion; greater resource-acquisition ability *appears* to increase attention for men and women in a similar manner, though upon closer examination the rate of increase for men far exceeds that for women (Fig. 2). The marginal means also allowed us to determine to what extent resource-acquisition ability helped to compensate for the large sex difference. Men who had +1 *SD* in resource-acquisition ability received a similar number of IOI to women who were –1 *SD* in resource-acquisition ability (*p* = .79 following a Bonferroni correction).

## Can Country-Level Variance Be Explained Using Nation-Level Variables?

To examine this question, we added GNI, GDI, OSR, and NEET to our base model. Because of the limited number of countries, we did this process separately for each country-level variable. Each model also contained the cross-level interactions between the country-level variable and the individual-level variables of sex and resource-acquisition ability. Three-way interactions were also included. The inclusion of country-level variables produced an improved model in all cases (Table 2).

**Table 2:** Additional models including the country-level variables of 5-year average Gross National Income (GNI), Operational sex ratio (OSR), Gender Development (GDI), and proportion of the population not in education, employment, or training (NEET)

	GNI		OSR		GDI		NEET	
*Fixed effects*	*B*	*SE*	*B*	*SE*	*B*	*SE*	*B*	*SE*
Intercept	−0.36	0.05	−0.37	0.06	−0.35	0.05	−0.36	0.06
Premium account status†	1.01	<0.01	1.01	<0.01	1.01	<0.01	1.01	0.01
Time since last login†	−0.26	<0.01	−0.26	<0.01	−0.26	<0.01	−0.26	<0.01
Account age	0.66	<0.01	0.66	<0.01	0.66	<0.01	0.66	<0.01
Sex [Men = 0; Women = 1]	2.03	0.08	1.99	0.08	2.00	0.08	1.99	0.08
Resource-acquisition ability	0.26	<0.01	0.26	<0.01	0.26	<0.01	0.26	<0.01
Sex × Resource-acquisition ability	−0.11	<0.01	−0.11	<0.01	−0.11	<0.01	−0.11	<0.01
CLV	−0.01	0.04	0.02	0.05	0.02	0.05	0.03	0.05
CLV × Sex	−0.10	0.06	−0.06	0.07	−0.03	0.08	−0.18	0.07
CLV × Resource-acquisition ability	−0.01	<0.01	<−0.01	<0.01	<0.01	<0.01	<0.01	<0.01
CLV × Sex × Resource-acquisition ability	<0.01	<0.01	<−0.01	<0.01	<−0.01	<0.01	<0.01	<0.01
*Random effects*	Var.comp	*SD*	Var.comp	*SD*	Var.comp	*SD*	Var.comp	*SD*
Intercept	0.06	0.25	0.07	0.27	0.06	0.25	0.07	0.26
Sex	0.14	0.38	0.15	0.39	0.15	0.39	0.14	0.37
*Model fit*	AIC	BIC	AIC	BIC	AIC	BIC	AIC	BIC
	11,371,668	11,371,854	11,371,808	11,371,994	11,371,666	11,371,852	11,371,642	11,371,828

† = standardized; CLV = County-level variable

**Fig. 3:**
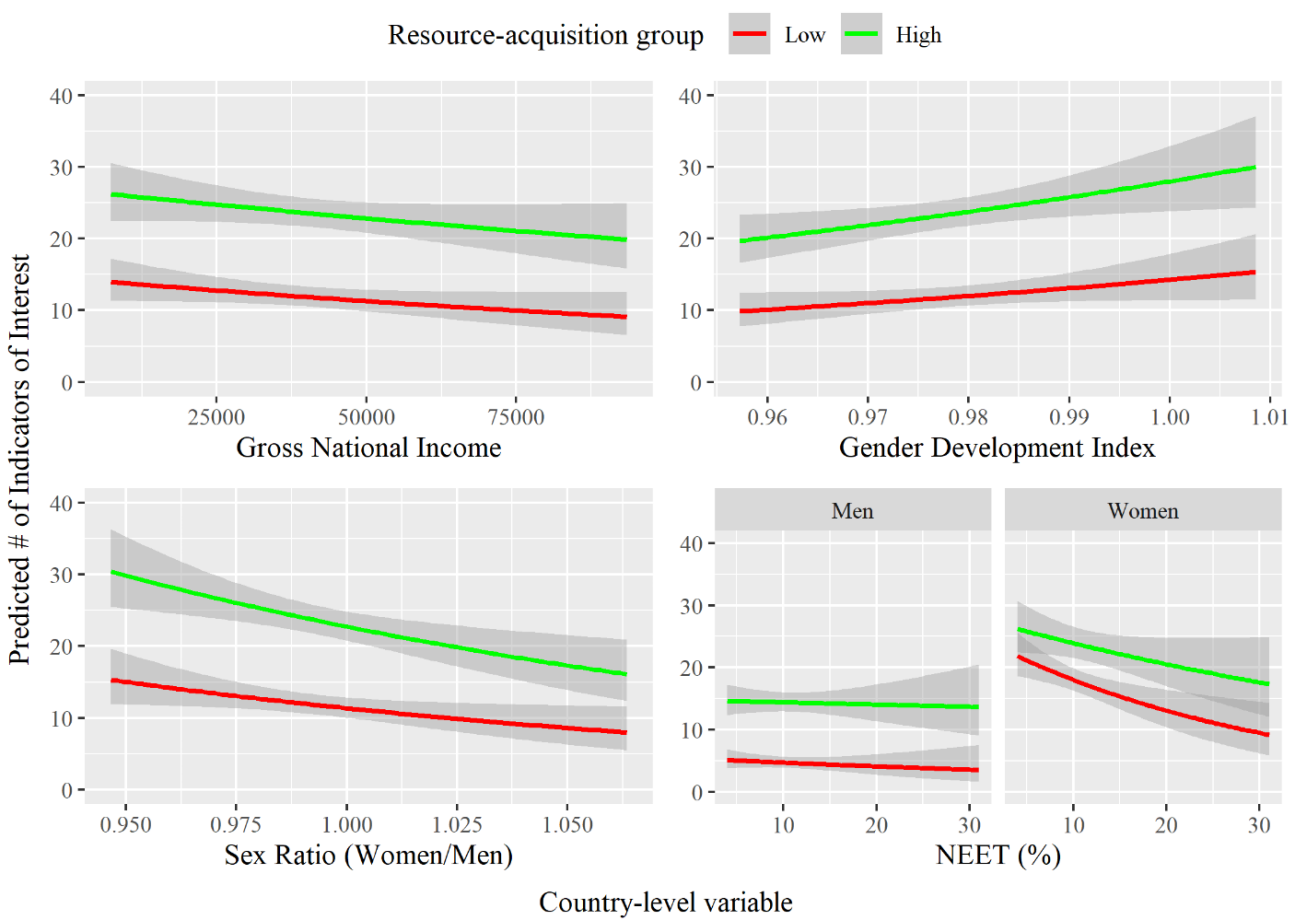
The predicted difference between high (+1 *SD*) vs. low (−1 *SD*) resource-acquisition ability on the level of interest a dating profile receives depending on the Gross National Income (GNI), Operational sex ratio (OSR), Gender Development (GDI), and proportion of the population not in education, employment, or training (NEET) of the country the profile sits in. Men and women are plotted separately for NEET due to the involvement of sex in the interaction. Lines are accompanied by ribbons showing 95% confidence intervals

We found that the effect of resource-acquisition ability was reduced in countries that were richer (GNI) and had more women of reproductive age than men (OSR), which was slightly enlarged in countries with greater gender equality (GDI). The effect of sex was not moderated by any of these country-level traits. The effect of resource-acquisition ability was also moderated by the proportion of the country not in education, employment, or training (NEET) but this differed for men and women. Resource-acquisition ability enhanced the attention of men’s more than women’s profiles, but this effect was exaggerated in countries with low unemployment. Note that our confidence in this effect was reduced at levels above 15% because only three countries in the sample (Mexico, South Africa, and Chile) had a NEET percentage above this (Fig. 3).

## Discussion

Using 1.8 M online dating profiles, we found that resource-acquisition ability and sex had a small, but robust influence on the amount of interest a dating profile received. Specifically, being a woman or having higher resource-acquisition ability led to increased numbers of messages, “winks,” and “likes” from other members (i.e., IOI). These patterns showed considerable cross-cultural consistency: resource-acquisition ability generally increased IOI in all countries, and, except for the USA, profiles of women generally received more IOI than those of men. Even in the USA, this sex difference reversed only at high levels of resource-acquisition ability (more than 2 *SD* above the mean). There was some variability between nations in the enhancing effect of resource-acquisition ability and its differential effect on the sexes, but this was simply a matter of degree. That is, resource-acquisition ability enhanced dating profile attention broadly, and for men more than women specifically, in all countries, though some more than others. These national sex differences accounted for only a slither of the total variance in attention received among the population. Nonetheless, we were still able to associate this variance with some country-level traits: gross national income, sex ratio, unemployment, and gender development.

Besides informing on the roles of culture, sex, and resource-acquisition ability on mate choice, we examined how differences in social, political, and economic differences in the various nations accounted for mate choice and sex differences therein. Although we cannot claim our results are definitive (given, e.g., sampling homogeneity in the countries), our results reveal that (1) sex differences persist in all countries sampled and (2) they appear relatively insensitive to the nation-level variables we considered. This may be more in line with evolutionary models of sex differences in mate choice than sociocultural ones because the latter treat sex differences as artifacts of culture (e.g., Eagly & Wood, 1999). Our results showed considerable agreement with studies from labs, smaller datasets, and mate preference research suggesting that even when people are actively choosing mates from the comfort of their couches, regardless of their country of origin, evolved mate selection tendencies are expressed.

Our research draws attention to the unique challenges of working with data of this magnitude (e.g., everything was significant). To cope with these challenges, we relied on confidence intervals to understand country-level patterns and descriptive differences (i.e., percent increase) as prima facie evidence of ostensible population-level effects. This process revealed just how small some of these effects might be. This may, in part, be the result of being unable to account for the primary feature that predicts romantic interest—physical attraction (Jonason & Antoon, 2019; Jonason et al., 2019; Kenrick et al., 1993; Li et al., 2002). At the same time, as both epidemiologists and evolutionary theorists have appreciated for some time, small effects over large populations and periods of time are not bereft of impact (Dawkins, 1996; Rose et al., 2008). In the online dating space, one additional message received might, for some people, change their mating trajectory entirely, with real consequences for their happiness and their reproductive success. One fruitful approach to deal with these kinds of data in the future may be to adopt Bayesian analyses instead of null-hypothesis-testing procedures.

## Limitations and Conclusions

Despite the size and scope of our study, it still had several limitations. First, we focused only on two predictors of romantic interest even though our data have several more. We did so because of the exponentially increasing complexity afforded by including more variables, and instead of focusing on describing who gets more IOI, we focused on theory-testing of microscopic issues. Of all the variables we have, we felt that resource-acquisition ability was the timeliest (e.g., the rise of the topic of sapiosexuality), the most useful for considering mate choice in relation to two theoretical paradigms, and one that has applied implications for mate searching and child mortality (Egebark et al., 2021; Hopcroft, 2021). The magnitude of these data is simply too much to conscientiously allow for exploratory tests when everything is likely to be “significant” but unlikely to be meaningful. Subsequent studies will examine the effects of height, marital status, number of children, and more. We presented here the first of a series of studies relying on “real” and “really big” data to understand cross-cultural patterns in mate choice using those seeking mates and people’s bone fide interest in them, not some hypothetical interest.

Second, resource-acquisition ability, as a factor influencing mate choice, is likely to have several related indicators, such as ambition, social status/level, and earning capacity (Buss, 1989; Li et al., 2002). We were only able to examine two of them—treated as a single index—given the limitations of what was collected. Although the two may not fully represent the larger construct of competence or resource-acquisition ability as we envision them, we think the results are more than defensible given their alignment with theory and having, themselves, been used as indicators of research in the past (Egebark et al., 2021; Hopcroft, 2021; Jonason & March, 2021). Indeed, the moderate correlation between the two may be reflective of the fact that we have only two indicators on a much larger mate-choice determinant (along with potential error in that data).

Third, despite the cross-national nature of this data, our sample was still WEIRD. (Henrich et al., 2010). Although countries such as Chile and Mexico might not traditionally be considered “Western,” they are educated, industrialized, and rich enough to have online dating services and Internet access. This may have created some range restriction and limit our results to just the countries where the dating service operates. It remains to be seen whether these patterns would hold up in African, South American, and Asian nations. Nevertheless, if we take an evolutionary perspective, differences in countries are a matter of degree for local calibration; *Homo sapiens* are humans everywhere (Buss, 1989; Thomas et al., 2020).

A final limitation involves our ability to account for country-level variance. Members in our dataset came from 24 countries, which allowed us to develop an understanding of how consistently resource-acquisition ability affects dating profile attention and how much this varies from country to country. However, even with two dozen nations, this aspect of our analysis was underpowered. Thus, we took a conservative approach when adding them to the base model. In contrast, other studies on the idea of “evoked culture” tend to examine country-level effects while controlling for variables such as longitude and latitude (Gangestad et al., 2006), though arguably, even then, such analyses are underpowered. Including more countries, particularly from non-WEIRD nations, would help us draw firmer conclusions.

In conclusion, we have provided the most definitive answers yet to the questions of the role of resource-acquisition ability in mate choice, whether there are sex differences in that role, and what nation-level factors might account for national patterns overall and in the sexes. We showed that greater resource-acquisition ability leads to more dating profile interest in data from more than 1.8 million people living in 24 nations who use the services of an international, online dating company. While both sexes received a boost in interest when they had more resource-acquisition ability, the increase was almost 2.5 times stronger in men than in women. And last, resource-acquisition ability tended to be slightly less important in richer countries with more women of reproductive age than men, and slightly more important in cultures with greater gender equality. Higher levels of unemployment also seemed to make resource-acquisition ability more important, but this effect was restricted to the amount of attention women’s profiles received. The relative primacy and robustness of sex differences suggest evolutionary models of mate choice may be more powerful than sociocultural ones when it comes to resource-acquisition ability.

## Data Availability

A summary data file is provided on the Open Science Framework. The actual data are proprietary but can be shared given some legal considerations. If interested, contact the first author. A shared summary and country level data are available via OSF (https://osf.io/r5294/?view_only=87b563ad5a4d4fa3946f66258cda520d).

## Footnotes

1 https://en.wikipedia.org/wiki/Cost_of_raising_a_child#cite_note-1.

2
https://www.statista.com/statistics/1041693/united-states-all-time-child-mortality-rate/.

3 We admit there are potentially limitless numbers of nation-level variables to consider. Readers interested in exploring others are encouraged to contact the authors or extract values from the supplementary data (see note 4).

4
https://osf.io/r5294/?view_only=87b563ad5a4d4fa3946f66258cda520d.

5 Results were conceptually similar using these two independently but inferior in terms of AICs. Combining them reduces Type I error inflation and gets at a broader construct than either can on their own. Item-specific tests are also available on the OSF site for this project.

6
https://datacatalog.worldbank.org/.

7
http://hdr.undp.org/en/content/gender-development-index-gdi.
